# Non-surgical facial harmonization for gender affirmation and psychosocial well-being in transmasculine persons: an exploratory mixed-methods study

**DOI:** 10.1590/1516-3180.2025.3274.25022026

**Published:** 2026-06-29

**Authors:** Liliane Lins-Kusterer, Victor Augusto Bastos e Silva, João Gabriel Macedo Briglia, José Valber Lima Meneses, Larissa do Nascimento Sampaio Celestino, Iza Maura Alves Travenzoli, Rodrigo Fernandes Weyll Pimentel

**Affiliations:** I Maxillofacial Surgeon, Departamento de Medicina Preventiva e Social (DMPS), Faculdade de Medicina da Bahia, Universidade Federal da Bahia (FMB-UFBA), Salvador (BA), Brazil.; II Undergraduate student. Faculdade de Medicina da Bahia, Universidade Federal da Bahia (FMBUFBA), Salvador (BA), Brazil.; III Undergraduate student. Faculdade de Medicina da Bahia, Universidade Federal da Bahia (FMBUFBA), Salvador (BA), Brazil.; IV Departamento de Cirurgia, Faculdade de Medicina da Bahia, Universidade Federal da Bahia (FMB-UFBA), Salvador (BA), Brazil.; V Hospital Dentistry Resident, Hospital Universitário Professor Edgard Santos (HUPES), Faculdade de Medicina da Bahia, Universidade Federal da Bahia (FMB-UFBA), Salvador (BA), Brazil.; VI Hospital Dentistry Resident, Hospital Universitário Professor Edgard Santos (HUPES), Faculdade de Medicina da Bahia, Universidade Federal da Bahia (FMB-UFBA), Salvador (BA), Brazil.; VII Departamento de Saúde da Família, Faculdade de Medicina da Bahia, Universidade Federal da Bahia (FMB-UFBA), Salvador (BA), Brazil.

**Keywords:** Transmasculine persons, Gender-affirming care, Facial recognition, Hyaluronic acid, Health promotion, Facial masculinization, Gender congruence, Non-surgical gender affirmation, Transgender health, Body image satisfaction, Psychosocial outcomes

## Abstract

**BACKGROUND:**

Facial appearance plays a central role in gender recognition and identity congruence, particularly among transmasculine individuals. Although the demand for gender-affirming care has increased, evidence regarding the psychosocial implications of nonsurgical facial masculinization using hyaluronic acid remains limited, especially within public health systems.

**OBJECTIVES:**

To describe the subjective and psychosocial experiences related to nonsurgical facial harmonization with hyaluronic acid among transmasculine individuals receiving care in public health settings.

**DESIGN AND SETTING:**

A mixed-methods exploratory study with a qualitative core component was conducted in a public, gender-affirming outpatient clinic in Salvador.

**METHODS:**

Six transmasculine patients participated in semi-structured interviews. The FACE-Q Satisfaction with Facial Appearance Overall Scale (SFAOS) and Beck Anxiety Inventory (BAI) were administered before and 30 days after the procedure as complementary quantitative measures. Qualitative data were analyzed using thematic content analysis based on Bardin’s framework, with sampling guided by theoretical saturation.

**RESULTS:**

Participants reported perceived reductions in facial dysphoria, improvements in self-perception, and greater social confidence after the procedure. Complementary quantitative measures indicated consistent short-term increases in facial satisfaction scores and a trend toward reduced anxiety.

**CONCLUSIONS:**

In this exploratory study, nonsurgical facial harmonization with hyaluronic acid was associated with short-term improvements in self-perceived facial satisfaction and psychosocial comfort among transmasculine participants. Given the small sample size and short follow-up period, these findings should be interpreted as preliminary. Larger studies with longer follow-up periods and comparative designs are warranted to evaluate the durability and generalizability of our findings.

## INTRODUCTION

 Facial harmonization, traditionally associated with aesthetic enhancement in cisgender populations, has become an increasingly relevant strategy in transgender healthcare, especially for individuals seeking gender-affirming care. For transmasculine persons, the face constitutes a critical site in the gender affirmation process because of its prominent role in social gender perception. Masculine-coded features such as a broad jawline, projected chin, and balanced facial proportions are strongly tied to cisnormative ideals and often become sources of dysphoria for individuals whose appearance diverges from these standards.^1,2^ Recent findings underscore the centrality of facial appearance for transgender and non-binary individuals who report specific preferences and expectations tied to aesthetic procedures as part of their identity expression.^3^

 Although testosterone therapy significantly influences body composition, muscle mass, and voice pitch, its effects on craniofacial bone structure remain limited, leaving many transmasculine persons with facial features that do not align with their gender identity.^2,4^ In this context, non-surgical interventions, such as injectable hyaluronic acid fillers, have gained attention as viable alternatives to orthognathic or implant-based procedures. These treatments offer not only aesthetic enhancement but also meaningful subjective impacts on self-image, self-esteem, and social integration.^ 5,6^

 The growing use of hyaluronic acid fillers for facial masculinization demonstrates their relevance in gender-affirming care. Fillers enable structural modifications to the jaw, chin, and midface with minimal risk, reduced downtime, and reversibility, factors that are especially valued by individuals seeking gradual and customizable transitions.^5^ Rheological advancements have allowed clinicians to tailor product selection to skin thickness and target anatomical depth, further enhancing safety and precision.^7^

 Despite these advances, transmasculine persons continue to face invisibility in terms of public policy and academic research. Much of the literature focuses on transfeminine persons, perpetuating a knowledge gap regarding specific needs, mental health outcomes, and healthcare barriers experienced by transmasculine individuals.^1,2^ Recent integrative reviews emphasize the need to broaden the scope of gender-affirming care to include nonsurgical approaches as essential elements in the promotion of psychosocial well-being and identity affirmation.^4,5^

 In this context, the present study aimed to describe subjective and psychosocial experiences related to nonsurgical facial harmonization with hyaluronic acid as a gender-affirming procedure in transmasculine persons. 

## METHODS

### Study design and setting

 This was a mixed-method exploratory study with a qualitative core component and complementary quantitative pre- and post-assessments. The qualitative component followed a descriptive and interpretative approach aimed at understanding the experiences of transmasculine individuals undergoing nonsurgical facial masculinization. The quantitative component assessed short-term changes in anxiety symptoms and satisfaction with the facial appearance. 

 This study was conducted at the Gender Facial Affirmation Outpatient Clinic of Hospital Universitário Professor Edgard Santos (HUPES), Faculdade de Medicina da Bahia of the Universidade Federal da Bahia (FMB-UFBA), Salvador, between March and June 2025. 

### Participants and sampling

 Six transmasculine adults were recruited from among outpatient service users through purposive sampling. Eligible participants were individuals who self-identified as transmasculine, aged 18 years or older, had undergone continuous masculinizing hormone therapy for at least 2 years, and had not previously undergone facial harmonization procedures. All of the participants who met these criteria and agreed to participate were included in the study 

 The sampling process followed the criteria of theoretical and thematic saturation, a methodological principle in qualitative research, whereby data collection is concluded when no new themes or relevant insights emerge from subsequent interviews. This approach ensured that the gathered material was sufficient to deeply explore the research questions and support a consistent and meaningful thematic analysis. Saturation was monitored continuously during the interview process. Once the narratives began to repeat key patterns without adding new dimensions, the research team determined that the saturation point had been reached, validating the adequacy of the sample size for the exploratory goals.^8^

### Procedure

 Facial masculinization was performed using a standardized hyaluronic acid injection protocol. A total of 3 mL was administered bilaterally, with 0.5 mL supraperiosteal bolus injections placed at each mandibular angle and the chin. The injections, consisting of hyaluronic acid with lidocaine (Restylane Lyft ^®^, Galderma), were delivered using a needle directly on the periosteum to enhance projection and angular definition. 

 Along the mandibular body, at the level of the second lower premolar, a 22G × 50 mm cannula was introduced through a single entry point. A retrograde linear threading technique was performed in a deep plane, delivering 0.5 mL of Restylane Define^®^ (Galderma) per side to improve mandibular contour continuity. 

 All procedures were performed by the same experienced surgeon using identical anatomical landmarks and injection techniques. Specific commercial products are mentioned solely for technical reproducibility, and do not imply endorsements. 

### Data collection and definitions

 Data were collected through individual semi-structured interviews conducted in a private setting either immediately after the procedure or during follow-up appointments. The interview guide explored perceptions of facial appearance, psychosocial impact of the intervention, and experiences related to gender dysphoria, selfesteem, institutional support, and access to public policy. 

 The Satisfaction with Facial Appearance Overall Scale (SFAOS), a subscale of the FACE-Q,^9^ is a psychometrically validated tool developed to assess how satisfied individuals are with the general appearance of their faces. The SFAOS consists of a set of items that prompt participants to rate their satisfaction with facial attributes, such as symmetry, balance, and overall appearance, at different times of the day. Responses were collected using a 4-point Likert scale ranging from “very dissatisfied” to “very satisfied.” This quantitative approach complements the qualitative data by providing a structured measure of subjective appearance-related outcomes.^9^

 The Beck Anxiety Inventory (BAI) is a widely used, psychometrically validated instrument designed to measure the severity of anxiety symptoms in clinical and research settings. It consists of 21 self-report items that describe common somatic and cognitive symptoms of anxiety, such as numbness, dizziness, fear of the worst occurrence, or difficulty in breathing. Each item was rated on a 4-point scale ranging from 0 (“not at all”) to 3 (“severely”), referring to how much the symptom bothered the respondent during the past week. The total score ranges from 0 to 63, with interpretive guidelines classifying the scores into minimal (0–10), mild (11–19), moderate (20–30), and severe (31–63) anxiety levels. This tool allows for a nuanced understanding of emotional states that may intersect with gender dysphoria and identity affirmation processes.^10^

### Statistical analysis

 Quantitative data were analyzed descriptively to assess within-participant changes between baseline and 30-day follow-up. Mean change scores (Δ) were calculated as 30-day score minus baseline score for both the BAI and the FACE-Q Satisfaction with SFAOS. The 95% confidence intervals (CI) for the mean paired differences were calculated to estimate the precision of the change. Standardized mean change was calculated using Cohen’s dz (dz = t/√n) to quantify the magnitude of within-participant effects. Given the small sample size and exploratory design, emphasis was placed on magnitude and precision rather than on confirmatory hypothesis testing. Analyses were performed using SPSS software (version 18.0; SPSS Inc., Chicago). 

### Qualitative analysis

 All interviews were audio-recorded, fully transcribed, and analyzed using thematic content analysis according to Bardin’s framework.^8^ This methodological approach consists of three structured phases: (1) pre-analysis, involving a floating reading of the material and constitution of the corpus; (2) exploration of the material, including identification of meaning units, coding, and progressive categorization; and (3) treatment and interpretation of results, in which themes were consolidated and analytically articulated. 

 Interviews were conducted sequentially and the analysis was conducted concurrently with data collection. After each interview, the transcripts were read in full and initially coded to identify recurring meaning units related to facial perception, dysphoria, psychosocial impact, and institutional experiences. The codes were iteratively refined through constant comparisons across interviews, leading to the consolidation of thematic cores. 

 Thematic saturation-guided recruitment was operationally defined as the point at which subsequent interviews no longer generated new codes or conceptual categories, and the thematic structure remained analytically stable. 

 Coding and categorization were conducted by the primary researcher with iterative discussions of emerging themes within the research team to enhance interpretive coherence and analytical transparency. 

 To ensure methodological rigor and transparency in qualitative reporting, this study followed the Consolidated Criteria for Reporting Qualitative Research (COREQ).^11^

### Ethical considerations

 The study adhered to all ethical principles outlined in Resolution No. 466/2012 of the Brazilian National Health Council and was approved by the Research Ethics Committee of the Universidade Federal da Bahia (UFBA) (approval number 6.469.528). 

## RESULTS

 Six transmasculine individuals participated in the study. The mean age was 34.17 years (SD = 12.49). Five participants were single and one reported being in a relationship. Four had completed higher education and two had completed high school. Five participants were employed, but all reported monthly incomes below the Brazilian minimum wage. Four participants reported engaging in regular physical activity, and one (16.7%) was identified as a smoker. All participants engaged in regular psychological follow-up at the time of the study. One participant had a previous diagnosis of severe anxiety disorder. 

### Anxiety symptoms

 The mean BAI score decreased from 11.33 (SD = 10.84) at baseline to 8 (SD = 6.72) after 30 days. The mean within-participant change (Δ) was −3.33 points, corresponding to a standardized mean change (Cohen’s dz) of 0.77, indicating a moderate-to-large within-participant reduction in anxiety symptoms. 

 Although variability across participants was observed, the overall direction of change was toward lower anxiety at follow-up. Considering the limited sample size and short-term assessment, these findings were interpreted as exploratory rather than confirmatory evidence. 

### Satisfaction with facial appearance

 Consistent within-participant improvements were observed across all FACE-Q satisfaction scores in the facial appearance domains at 30 days (**Table 1**). The mean change scores (Δ) ranged from +1.17 to +2.17 points, indicating higher satisfaction at follow-up compared with baseline. 

**Table 1:** Within-participant changes in FACE-Q satisfaction with facial appearance domains from baseline to 30 days (n = 6)

**Domain**	**Baseline Mean (SD)**	**30-Day Mean (SD)**	**Mean Change (Δ)**	**95% CI of Δ**	**Cohen’s dz**
Facial symmetry	1.5 (0.55)	3.17 (0.75)	+1.67	0.58–2.75	1.61
Facial harmony	1.5 (0.55)	3.33 (0.82)	+1.83	0.61–3.06	1.57
Facial proportion	1.5 (0.55)	3.17 (0.75)	+1.67	0.58–2.75	1.61
End-of-day appearance	1.17 (0.41)	3 (1.1)	+1.83	0.8–2.87	1.86
Freshness	1.83 (0.98)	3 (1.1)	+1.17	0.14–2.2	1.19
Rested appearance	1.67 (0.82)	2.83 (0.98)	+1.17	0.14–2.2	1.19
Profile view	1.5 (0.55)	2.83 (0.98)	+1.33	0.48–2.19	1.63
Appearance in photographs	1.17 (0.41)	3.33 (0.52)	+2.17	1.74–2.6	5.31

Values are presented as means (standard deviations). Mean change (Δ) was calculated as 30-day score minus baseline score. Positive Δ values indicate higher satisfaction at follow-up. Δ = mean within-participant change (30-day minus baseline). 95% CI = 95% confidence interval of the mean paired difference. Cohen’s dz represents the standardized mean change for paired samples (dz = t/√n). Effect sizes were reported to describe the magnitude of change in this exploratory study.

 The standardized mean changes (Cohen’s dz) ranged from 1.19 to 5.31, reflecting substantial within-participant effects across domains. The largest standardized change was observed in the “appearance in photographs” domain, reflecting a highly consistent within-participant improvement. 

 Given the small purposive sample and exploratory design, effect sizes were presented to describe the magnitude of within participant changes rather than to support confirmatory inference. 

**Figure 1:**
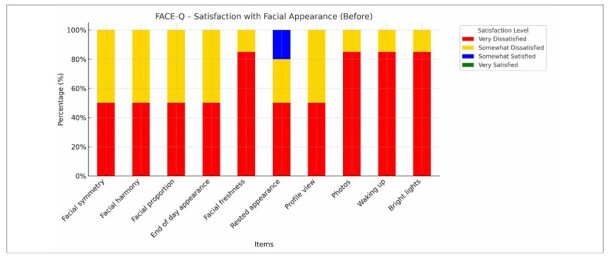
Distribution of satisfaction levels with facial appearance before the procedure according to the FACE-Q instrument (n = 6).

 Across domains, descriptive inspection of response distributions demonstrated a shift from predominantly dissatisfaction categories at baseline to predominantly “somewhat satisfied” or “very satisfied” responses at 30 days (**Figures 1** **and** **2**). 

**Figure 2:**
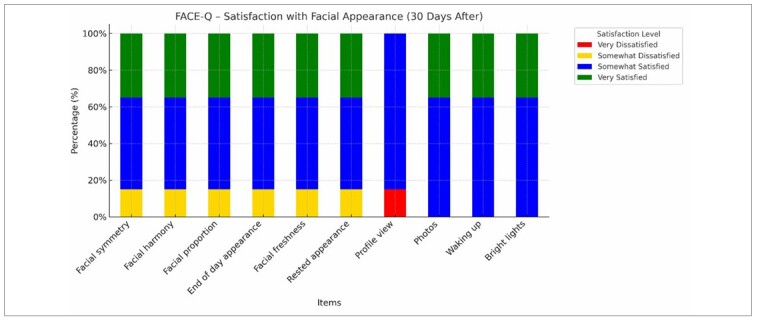
Distribution of satisfaction levels with facial appearance 30 days after the procedure according to the FACE-Q instrument (n = 6).

 From the initial skimming and content coding of the interviews, five thematic cores emerged to structure the analysis: (1) facial dysphoria and self-perception; (2) impact on self-esteem and social relationships; (3) support and care during the transition process; (4) minimally invasive procedures as an alternative to surgery; and (5) invisibility of transmasculine persons in public policies and within the Brazilian Unified Health System (SUS). These themes served as the basis for the qualitative analysis and are presented in **Table 2**. 

**Table 2:** Thematic cores, descriptions, and illustrative quotes from participants

**Thematic core**	**Analytical description**	**Illustrative quotes**
Facial dysphoria and self-perception	Dissatisfaction with specific facial features – particularly the jawline, chin, and facial symmetry – emerged as a core marker of dysphoria. Participants expressed emotional discomfort when seeing themselves in the mirror, with aesthetic incongruities reinforcing the distress associated with gender dysphoria. Subtle facial adjustments were described as directly alleviating this discomfort.	“Every time I looked in the mirror, I felt really uncomfortable. Mainly because of my jaw asymmetry.”“ The chin area always made me feel very insecure, especially in photos.” “My jaw was crooked, and now I can see both sides in a harmonious way.”
Impact on self-esteem and social relationships	Participants reported increased self-confidence and improved social interactions after the procedure. They began to feel more comfortable in public spaces, in photographs, and in interpersonal relationships. Being perceived by others in a way that aligned with their gender identity significantly contributed to their psychological well-being.	“After the procedure, I felt more confident going out, talking to people, looking them in the eye.” “I stopped avoiding photos and started feeling more like myself in social settings.” “People started saying I looked more masculine, and that made me feel really good.”
Support and care during the transition	The availability of supportive relationships – whether familial, peer-based, or institutional – was pivotal during the transition process. Emotional and psychological support, as well as respectful and attentive care, were experienced as affirming and therapeutic. The procedure itself was described not just as clinical but as a form of care and affection.	“I have support from my family, I receive psychological counseling, and I use hormones regularly.” “I didn’t receive support during my transition, but I found help from friends.” “There’s general resistance from my family, but my mother supported me.” “This care felt like a gesture of affection to me.”
Minimally invasive procedures as an alternative to surgery	All participants endorsed the hyaluronic acid procedure as a safe and effective option. Its non-permanent and reversible nature was valued, especially for those who were not ready for surgical interventions. These procedures were framed not as superficial beautification, but as tools for authentic identity expression and improved mental health.	“I think it’s a faster, less painful process, and the result is amazing.” “This type of procedure can help a lot of people.” “It may seem like a small thing to others, but this small detail completely changed our self-esteem.” “Beyond improving my self-esteem, this procedure made me feel seen.”
Invisibility of transmasculine persons in public policies and the Brazilian Unified Health System	Participants highlighted the systemic invisibility of transmasculine persons in health policies and public narratives. Many reported mental health challenges linked to social marginalization. They emphasized that access to aesthetic procedures within the public health system would not only improve individual well-being but also symbolize broader social recognition of transmasculine identities.	“Transmasculine persons are an invisible group within the trans population.” “It’s common among us to experience anxiety, stress, and depression.” “We transmasculine persons go through violent situations that often don’t make the news.” “This isn’t aesthetics for vanity. It’s identity affirmation. It’s mental health.” “This procedure is necessary because the person needs it for their affirmation.”

## DISCUSSION

 The findings of this exploratory study suggest that nonsurgical facial harmonization with hyaluronic acid may be associated with short-term improvements in self-perceived facial satisfaction and psychosocial comfort among transmasculine participants. For the quantitative component, within-participant change estimates indicated consistent improvements across the FACE-Q satisfaction domains and a trend toward reduced anxiety symptoms. These quantitative results are presented as complementary magnitude estimates (Δ, 95% CI: and standardized mean change) rather than confirmatory evidence, and are interpreted alongside qualitative narratives that contextualize perceived changes in dysphoria-related distress, self-esteem, and social confidence. This aligns with existing literature that highlights the face as a central element in gender recognition and identity validation, especially among transmasculine individuals.^3^ Although anxiety scores showed a trend toward reduction, variability was observed and changes should be interpreted with caution. Taken together, the qualitative narratives and exploratory quantitative change estimates suggest that minimally invasive facial interventions may contribute to perceived psychosocial comfort and identity congruence in the short-term. Given the limited sample size and follow-up period, these findings should be understood as preliminary and hypothesis-generating rather than confirmatory. 

 The findings of this study suggest that nonsurgical facial harmonization using hyaluronic acid may be associated with perceived improvements in emotional well-being, self-esteem, and identity congruence among transmasculine persons. Facial dysphoria, particularly concerning the jawline, chin, and facial symmetry, emerged as a central source of psychological distress in participants’ narratives, aligning with international evidence that underscores the face as a critical site for gender dysphoria, owing to its influence on social gender recognition.^2,5^

 These results are consistent with those of recent studies that describe nonsurgical procedures as promising or potentially useful approaches within gender-affirming care, particularly for transmasculine individuals who face barriers to surgical access or prefer less invasive alternatives.^1,6^ In this context, the incorporation of such practices into public health systems may align with the principles of autonomy, accessibility, and equity in gender-affirming care. 

 The high levels of dissatisfaction captured by the FACE-Q instrument were consistent with the participants’ narratives and echo findings in the literature, showing that while hormone therapy induces systemic bodily changes, it has a limited impact on craniofacial bone structure. In this context, injectable fillers, particularly hyaluronic acid, have been described as minimally invasive and adjustable options that allow gradual and individualized facial masculinization.^2,6^

 Participants described perceived improvements in self-esteem following the procedures, as reflected in their verbal reports, and reported changes in daily behavior such as increased comfort with being photographed and engaging socially. These observations are consistent with those of previous studies suggesting associations between appearance-related changes and self-image or dysphoriarelated distress.^1,6^ Some participants also reported reduced anxiety and enhanced feelings of safety, which may indicate a link between facial harmonization and perceived psychological well-being, an area that remains underexplored in research involving transmasculine populations. 

 Institutional and familial support emerged as important contextual elements in participants’ experiences. Those who reported supportive relationships with either family members or healthcare teams reported more positive and affirmative transition processes. These narratives highlight the importance of healthcare environments that are not only technically competent, but also emotionally responsive and affirming.^1^

 A preference for minimally invasive procedures over surgical options was evident in participants’ narratives. They emphasized the reversibility, adjustability, and natural-appearing results associated with hyaluronic acid injections, aspects that align with patientcentered care principles that prioritize autonomy and individualized trajectories of gender affirmation.^1,2^ The possibility of gradual, non-permanent changes was described as particularly meaningful for individuals who wish to adapt their appearance over time. Such flexibility may accommodate evolving gender expression and reduce concerns related to the permanence of surgical interventions, features valued by the participants in this study. 

 From an anatomical perspective, the lower third of the face plays a prominent role in gender recognition, particularly in relation to the mandibular contour and chin projection. The clinical literature describes the use of hyaluronic acid and other dermal fillers as minimally invasive approaches to enhance the mandibular angle definition and lower facial projection in transgender patients.^5,6^ These techniques have been reported as strategies for facial masculinization through targeted augmentation of the mandibular body and chin. In our study, the participants’ narratives indicated that modifications in the jawline and chin were especially meaningful in everyday contexts, such as photographs and social interactions, contributing to a perceived sense of gender congruence. The adjustability and reversibility of injectable fillers are advantageous, particularly for individuals who are navigating evolving gender expressions or facing structural barriers to surgical access. In public health settings, where access to surgery may be limited, such minimally invasive options may represent an additional pathway within gender-affirming care, reinforcing the importance of individualized and gender-sensitive approaches in aesthetic and dermatological practice. 

 Some studies have suggested that transgender men may present more varied or less predictable preferences regarding facial appearance than other gender-diverse populations.^3^ In this context, preferences may favor more neutral or less strongly gendered features, reflecting the complexity and heterogeneity of gender identity experiences. Within this framework, the adaptability of injectable fillers such as hyaluronic acid may be perceived as advantageous because they allow for gradual and individualized modifications aligned with patient-defined goals. 

 These findings are consistent with an integrative review^4^ that described associations between nonsurgical procedures and improvements in self-confidence and quality of life among transgender individuals. The review also emphasizes the limited availability of robust clinical evidence and highlights the need for clearer guidance and best-practice frameworks to inform the responsible implementation of these techniques. 

 Recent clinical studies have described treatment considerations for facial masculinization in transgender patients using injectable fillers.^2^ These reports illustrate how nonsurgical procedures such as hyaluronic acid injections have been applied to enhance mandibular angle definition and lower facial projection. In the cited clinical cases, volumizing fillers were used to achieve a broader and more angular contour consistent with patients’ aesthetic goals.^2^ While our study was exploratory and qualitative in nature, participants’ narratives describing increased comfort with their appearance and reduced dysphoria were consistent with these clinical observations. The non-permanent and adjustable nature of injectable treatments may offer flexibility to individuals seeking gradual and personalized gender-affirming modifications. 

 By situating our qualitative findings within the existing international clinical literature, nonsurgical facial harmonization can be understood as a potentially relevant component of genderaffirming care that carries both technical and psychosocial dimensions. Although exploratory, the participants’ accounts suggest that access to such interventions in public healthcare settings may hold symbolic and experiential significance beyond aesthetic changes. 

 Narratives on the relative invisibility of transmasculine individuals in public health policies highlight broader structural challenges within the Brazilian Unified Health System (SUS). While our findings cannot inform policy directly, they underscore the importance of continued research and dialogue regarding the scope and organization of gender-affirming services, including non-surgical approaches. 

### Limitations

 This study should be interpreted within the context of an exploratory mixed-method design. The small purposive sample (n = 6), recruited from a single public gender-affirming outpatient clinic, may limit the transferability to other healthcare settings or populations. However, the qualitative core component prioritized the depth of lived experience and contextual understanding over statistical representativeness, which was consistent with its exploratory objectives. 

 The absence of a control group and a 30-day follow-up restrict conclusions regarding long-term durability or comparative effectiveness. The quantitative component was intentionally designed to be complementary and descriptive, focusing on the withinparticipant magnitude of change and precision estimates rather than confirmatory hypothesis testing. These short-term quantitative indicators were integrated with qualitative narratives to provide contextualized interpretations rather than causal inferences. 

 Although all participants were engaged in ongoing psychological follow-up and only one reported a prior diagnosis of a more severe anxiety disorder, transition-related and clinical variables, such as the duration of social transition or prior gender-affirming procedures, were not systematically collected in a standardized form and may have contributed to individual variability in experiences. Future studies may benefit from incorporating a more structured characterization of these factors. 

 The analytic procedures followed Bardin’s structured content analysis framework, with explicit operationalization of thematic saturation and concurrent analysis. Within this epistemological approach, rigor was supported through systematic categorization and transparent reporting of the analytic phases. 

 Despite these limitations, this study represents one of the first structured empirical investigations of non-surgical facial masculinization conducted within a public healthcare context in Brazil. Integrating qualitative narratives with exploratory quantitative change estimates contributes contextually grounded evidence to an underexplored domain of transmasculine health and offers a foundation for larger, longitudinal, and comparative investigations. 

## CONCLUSION

 In this exploratory study conducted in a public gender-affirming outpatient clinic, transmasculine participants reported reduced facial dysphoria, improved self-perception, and greater social confidence following nonsurgical facial harmonization with hyaluronic acid. Complementary quantitative measures showed consistent short-term improvements in facial satisfaction domains and a trend toward reduced anxiety symptoms. 

 Given the small sample size, the absence of a control group, and the 30-day follow-up, these findings should be interpreted as preliminary and context-specific. Nevertheless, they highlight the potential psychosocial relevance of minimally invasive facial interventions within broader gender-affirming care pathways. Future studies with larger sample sizes, longer follow-up periods, and comparative designs are required to evaluate the durability, safety, and generalizability of the results. 

## Data Availability

The datasets generated and/or analyzed during the current study are not publicly available due to ethical and privacy restrictions involving sensitive patient information. Data may be available from the corresponding author, Liliane Lins-Kusterer, upon reasonable request and with approval from the appropriate ethics committee.

## References

[1] Dhingra N, Bonati LM, Wang EB, Chou M, Jagdeo J (2019). Medical and aesthetic procedural dermatology recommendations for transgender patients undergoing transition. J Am Acad Dermatol.

[2] De Boulle K, Furuyama N, Heydenrych I (2021). Considerations for the use of minimally invasive aesthetic procedures for facial remodeling in transgender individuals. Clin Cosmet Investig Dermatol.

[3] Cronin BJ, Fadich S, Lee JC (2024). Assessing preferences of facial appearance in transgender and gender nonbinary patients. Aesthetic Plast Surg.

[4] Lins-Kusterer L, Azevedo JF, Carvalho FM (2025). Non-surgical procedures for facial gender reassignment: integrative review. Braz J Hea Rev.

[5] Ascha M, Swanson MA, Massie JP (2019). Nonsurgical management of facial masculinization and feminization. Aesthet Surg J.

[6] Wu GT, Wong A, Bloom JD (2023). Injectable treatments and nonsurgical aspects of gender affirmation. Facial Plast Surg Clin North Am.

[7] Fagien S, Bertucci V, von Grote E, Mashburn JH (2019). Rheologic and physicochemical properties used to differentiate injectable hyaluronic acid filler products. Plast Reconstr Surg.

[8] Vaismoradi M, Turunen H, Bondas T (2013). Content analysis and thematic analysis: implications for conducting a qualitative descriptive study. Nurs Health Sci.

[9] Klassen AF, Cano SJ, Schwitzer JA, Scott AM, Pusic AL (2015). FACE-Q scales for health-related quality of life, early life impact, satisfaction with outcomes, and decision to have treatment: development and validation. Plast Reconstr Surg.

[10] Kabacoff RI, Segal DL, Hersen M, Van Hasselt VB (1997). Psychometric properties and diagnostic utility of the Beck Anxiety Inventory and the StateTrait Anxiety Inventory with older adult psychiatric outpatients. J Anxiety Disord.

[11] Tong A, Sainsbury P, Craig J (2007). Consolidated criteria for reporting qualitative research (COREQ): a 32-item checklist for interviews and focus groups. Int J Qual Health Care.

